# Supplementary material for: Clinical Impact of Immune Checkpoint Inhibitor (ICI) Response, DNA Damage Repair (DDR) Gene Mutations and Immune-Cell Infiltration in Metastatic Melanoma Subtypes
Source: Med Sci (Basel). 2022 May 24;10(2):26. doi: 10.3390/medsci10020026 (PMC9230974; doi:10.3390/medsci10020026)
Supplement: Supplementary file 1 [file medsci-10-00026-s001.zip › medsci-1650480-supplementary.pdf]

Table S1: Patient information and detected mutations.

Study ID	Age at metastatic diagnosis	Diagnosis/Tumour	Primary site	Melanoma subtype	Surgical/metastatic site	Detected mutations from panel	Rounds of ICI	Chemo/targeted therapy	PFS (mo)	OS (mo)
**Mel-I001**	55 Years	Malignant Melanoma	Scalp	HNM	Radical Neck dissection	BRAF p.V600E; BRAF p.G469R; ERBB2 p.S442L	IPILIMUMAB	Dabrafenib/Trametinib	2.9	40.5
**Mel-I002**	59 Years	Malignant Melanoma	Left pinna	HNM	Left lung, upper lobe	MYCL p.D198N; NRAS p.Q61H; PIK3R1 p.Q572*	IPILIMUMAB		1.5	10.0
**Mel-I003**	58 Years	Malignant Melanoma	Rigth calf	CM	Right leg calf	NRAS p.Q61R; CDKN2A p.R80*	IPILIMUMAB		0.4	80.9
**Mel-I004**	44 Years	Malignant Melanoma	Left cheek	HNM	Popliteal right knee	NTRK1 p.E581K; RICTOR splice; PIK3R1 p.P211S; APC p.L407F; RAD50 p.Q508*; MET p.S663L; PRKDC p.3541S; PTEN p.Q171*; FGFR2 p.P595S; FGFR2 p.D138N; FGFR2 p.G80R; POLE p.P172S; BRCA2 p.S1261F; BRCA2 p.P2612S; , MAP2K3 p.E341K; NF1 p.W571*; NF1 p.V921L; RPTOR p.A274V; GNAS p.D17N; NF1 p.L1441fs	IPILIMUMAB; Pembrolizumab	DTIC	6.5	47.8

**Mel-I005**	78 Years	Ocular melanoma	Ocular	UM	Liver metastasectomy	ERBB3 p.S1083C; GNAQ p.Q209L; PRKDC p.S2511P; RB1 p.T12P	IPILIMUMAB		1.5	15.5
**Mel-I006**	73 Years	Malignant Melanoma (arcal lentiginous)	Left toe	AM/MM	Toe, Lymph Node	BRAF p.D594A; BRCA2 p.I27M; NRAS p.D33E	IPILIMUMAB	Dartmouth chemotherapy	7.4	27.7
**Mel-I007**	58 Years	Malignant Melanoma	Scalp	HNM	Scalp Lesion	GNA11 p.Q209L; KIT p.T380M	IPILIMUMAB		2.8	5.1
**Mel-I008**	60 Years	Malignant Melanoma	Elbow	CM	Skin, left elbow	APC p.P2540S; ATR p.Q1627*; BRAF p.V600K; DDR2 p.P389L; GNAS p.A446V; KDR p.G537K; MYC p.K304E; NF1 p.S1684F; SMO p.R291Q	IPILIMUMAB		9.1	50.7
**Mel-I009**	78 Years	Malignant Melanoma	Cutaneous	CM	Skin, left jaw, excision	ALK p.D602N; APC p.V1675A; ARID1A p.Q802*; BRAF p.D594N; CDKN2A p.R80*; FGFR4 p.C271Y; KRAS p.G12N; NF1 splice; NF1 p.Q1515*; PRKDC p.C2552R; RB1 p.W563L; ROS1 p.S79L; TSC2 p.L567P	IPILIMUMAB		0.5	1.8
**Mel-I010**	57 Years	Malignant Melanoma	Perianal skin	AM/MM	Perianal skin/mucosa	KIT p.L576P	IPILIMUMAB; Pembrolizumab		2.7	14.1

**Mel-I011**	61 Years	Malignant melanoma	Cutaneous, back	CM	Lymph node/skin excision	BRCA1 p.N1840S	IPILIMUMAB + NIVOLUMAB; IPILIMUMAB		16.7	20.1
**Mel-I012**	60 Years	Malignant melanoma	Left leg	CM	Left leg lesion	BCL6 p.R24C; CDKN2A splice; ERBB2 p.D1019N; NRAS p.Q61R; ROS1 splice	IPILIMUMAB		3.5	52.3
**Mel-I013**	62 Years	Ocular melanoma	Ocular	UM	Liver metastasectomy	ARID1A p.P912L; GNAQ p.Q209P	IPILIMUMAB		39.7	50.1
**Mel-I014**	70 Years	Malignant melanoma	Cutaneous, right leg	CM	Skin excision right leg	ATR p.R515C; CTNNB1 p.G575V; MTOR p.E1610K; NRAS p.Q61R	IPILIMUMAB; Pembrolizumab		3.5	27.4
**Mel-I015**	65 Years	Malignant melanoma	Face	HNM	Left neck mass excision	ARID1A p.S486F; ATM p.R1086C; BRAF p.L584F; BRCA2 p.S146F; BRCA2 p.F1634S; KDR splice; MAP2K1 p.F53L; MAP2K1 p.D67V; MET p.P1171L; MTOR p.P404L; PIK3CA p.E545K	IPILIMUMAB; Pembrolizumab		2.7	27.3
**Mel-I016**	64 Years	Ocular melanoma	Ocular	UM	Liver metastasectomy	GNAQ p.Q209P; MTOR p.R1009Q; PRKDC p.R1360del	IPILIMUMAB; Pembrolizumab	Dartmouth chemo	3.2	23.5
**Mel-I017**	62 Years	Malignant melanoma	Cutaneous, abdominal wall	CM	Wedge resection of lung	AR p.P555S; ARID1A p.P1456L; BCL6 p.P151S; BRD4 p.*723L; CDKN2A p.H98Y; GNAS p.P541L;	IPILIMUMAB; Pembrolizumab	Dartmouth chemo, DTIC	3.5	35.6

						KDR p.A1218T; NF! P.Q282*; NF1 p.R2517*; PALB2 p.I1180M				
**Mel-I018**	41 Years	Malignant melanoma	Right knee	CM	Skin, right knee excision	ARID1A p.P1469L; ARID1A p.T1470fs; BRAF p.V600E; MYCN p.S369N	IPILIMUMAB; Pembrolizumab	DABRAFENIB/TRAMETINIB; Dartmouth chemo	5.3	100.7
**Mel-I019**	61 Years	Malignant melanoma	Cutaneous	CM	Skin, right arm	NF1 p.L2073F; NRAS p.Q61R; POLE p.P172R; ROS1 p.R1035*	IPILIMUMAB; IPILMUMAB; Pembrolizumab		2.8	63.4
**Mel-I020**	68 Years	Malignant melanoma	Cutaneous, right leg	CM	Right groin node	BRCA2 p.G1761E; NRAS p.Q61K; NTRK1 p.V200G; ROS1 p.E2265D	IPILIMUMAB; Pembrolizumab		19.4	53.6
**Mel-I021**	62 Years	Ocular melanoma	Ocular	UM	Liver metastasectomy	BRD4 p.R326Q; GNAQ p.Q209L	IPILIMUMAB		2.8	10.7
**Mel-I023**	70 Years	Malignant melanoma	Radical neck dissection	HNM	Radical neck dissection	ALK p.G667E; ALK p.E405K; CDH1 p.V473I; CHEK2 p.R517G; GNAS p.P352F; GNAS p.P465S; HRAS p.G13E; KDR p.R1032Q; NF1 p.Q853*; NF1 p.R1241*; NF1 p.E1436K; NTRK1 p.D596N; POLD1 p.P1102S; PRKDC splice; RB1 p.Q35*; RB1 p.Q685*	PEMBROLIZUMAB		7.5	23.8

**Mel-I025**	70 Years	Malignant melanoma	Scalp	HNM	Liver metastasectomy	GNA11 p.Q209L	IPILIMUMAB; Pembrolizumab		3.4	177.7
